# Malaria vectors resistance to insecticides in Benin: current trends and mechanisms involved

**DOI:** 10.1186/s13071-015-0833-2

**Published:** 2015-04-12

**Authors:** Virgile Gnanguenon, Fiacre R Agossa, Kefilath Badirou, Renaud Govoetchan, Rodrigue Anagonou, Fredéric Oke-Agbo, Roseric Azondekon, Ramziath AgbanrinYoussouf, Roseline Attolou, Filemon T Tokponnon, Rock Aïkpon, Razaki Ossè, Martin C Akogbeto

**Affiliations:** Centre de Recherche Entomologique de Cotonou (CREC), Cotonou, Benin; Faculté des Sciences et Techniques de l’Université d’Abomey-Calavi, Abomey-Calavi, Benin; Université d’Agriculture de Kétou, Kétou, Benin; Programme National de Lutte contre le Paludisme, Cotonou, Benin

**Keywords:** Pyrethroids resistance, Bendiocarb resistance, Insensitive acetylcholinesterase-1, Knock-down resistance, Resistance map

## Abstract

**Background:**

Insecticides are widely used to control malaria vectors and have significantly contributed to the reduction of malaria-caused mortality. In addition, the same classes of insecticides were widely introduced and used in agriculture in Benin since 1980s. These factors probably contributed to the selection of insecticide resistance in malaria vector populations reported in several localities in Benin. This insecticide resistance represents a threat to vector control tool and should be monitored. The present study reveals observed insecticide resistance trends in Benin to help for a better management of insecticide resistance.

**Methods:**

Mosquito larvae were collected in eight sites and reared in laboratory. Bioassays were conducted on the adult mosquitoes upon the four types of insecticide currently used in public health in Benin. Knock-down resistance, insensitive acetylcholinesterase-1 resistance, and metabolic resistance analysis were performed in the mosquito populations based on molecular and biochemical analysis. The data were mapped using Geographical Information Systems (GIS) with Arcgis software.

**Results:**

Mortalities observed with Deltamethrin (pyrethroid class) were less than 90% in 5 locations, between 90-97% in 2 locations, and over 98% in one location. Bendiocarb (carbamate class) showed mortalities ranged 90-97% in 2 locations and were over 98% in the others locations. A complete susceptibility to Pirimiphos methyl and Fenitrothion (organophosphate class) was observed in all locations with 98-100% mortalities. Knock-down resistance frequencies were high (0.78-0.96) and similar between *Anopheles coluzzii*, *Anopheles gambiae*, *Anopheles arabiensis,* and *Anopheles melas*. Insensitive acetylcholinesterase-1 was rare (0.002-0.1) and only detected in *Anopheles gambiae* in concomitance with Knock-down resistance mutation. The maps showed a large distribution of Deltamethrin resistance, Knock-down mutation and metabolic resistance throughout the country, a suspected resistance to Bendiocarb and detection of insensitive acetylcholinesterase-1 from northern Benin, and a wide distribution of susceptible vectors to Pirimiphos methyl and Fenitrothion.

**Conclusion:**

This study showed a widespread resistance of malaria vectors to pyrethroid previously located in southern Benin, an early emergence of carbamates resistance from northern Benin and a full susceptibility to organophosphates. Several resistance mechanisms were detected in vectors with a potential cross resistance to pyrethroids through Knock-down and metabolic resistance mechanisms.

## Background

For malaria vector control intervention, Indoor Residual Spray and Insecticide Treated Net are so far the most effective tools used [1-3]. These two interventions are based on the use of different classes of insecticide.

Pyrethroid insecticides are considered most suitable for mosquito nets treatment due to their high insecticidal potency at low dosages and a relative safety for human contact and domestic handling [2]. They included: Lambdacyhalothrin, Permethrinn, Alpha-cypermethrin, Etofenprox, and Cylfluthrin [2]. They have a quick knock-down and lethal effect on *Anopheles gambiae sensu lato* (*An. gambiae s.l*) mosquitoes at low concentrations [3]. The majority of long-lasting insecticide-treated nets freely distributed in all localities in Benin through mass-distribution campaigns and routine distributions since 2007 were Deltamethrin and Permethrin based [4-6].

Bendiocarb was previously used for indoor residual spray in Oueme province in Benin from 2008 to 2010 (6 spray cycles) and showed a significant reduction in malaria transmission [7,8]. It is also used for indoor residual spray in Atacora province each year with one spray cycle per year since 2011 [9].

But the use of these vector control tools cannot be efficient if their use does not take into account the environmental factors associated with insecticide resistance before their implementation. Pyrethroids resistance was reported in several localities in southern Benin since 1999 [10-13] and represented also a threat for these interventions. In addition, the lack of baseline data before the implementation of vectors control intervention in an area needs to be addressed. For example, the National Malaria Control Program (NMCP) needs data on the dynamics of insecticide resistance in all the epidemiological patterns of the country, particularly a map on the entomological situation of Benin, to be used as a guide to the choice of vector control strategies. This study was developed in this framework. The study was conducted in eight randomly selected sites. The eight sites were selected in the main malaria eco-epidemiological areas of Benin where geography (climate, land, vegetation and agriculture practices) and malaria prevalence were different [14]. At each site, the level of vector resistance (Pyrethroids, Carbamates, and Organophosphates) and the resistance mechanisms (Kdr-west, Ace-1^R^ and metabolic resistance) involved were assessed. A mapping of the distribution of vector resistance and vector resistance mechanisms was done. Data gathered in the different malaria eco-epidemiological areas will guide malaria vectors resistance strategies in Benin.

## Methods

### Study design

The study was conducted in each rainy season (April-July; October-November) in 2012, 2013, and 2014 in eight districts randomly selected from different geo-epidemiological regions in Benin [14]. The selected districts were:

- Adjohoun, Allada, Pobe, and Ouidah in wet savanna and degraded forest regions, southern Benin [15-18];
- Kandi and Malanville in dry savanna region, northern Benin [19,20];
- Dassa in the central part, and Parakou in the north southern part, are located between the dry and wet savanna region (transition zone) [21,22].

Atacora region, where indoor residual spray was ongoing, was excluded due to the specific vector resistance monitoring system implemented in this area, that was well documented [9,23].

*Anopheles gambiae s.l* larvae were collected based on dipping method in four villages randomly selected in each district, then bred to adult phase (They were pooled together to have sufficient adult of the same physiological age by district) and tested with different classes of insecticides (with around 150 mosquitoes tested/insecticide/location) using WHO susceptibility (tube/cylinder) tests guidelines [14]. Mosquitoes tested were subjected to specific polymerase chain reaction treatments for species identification, and determination of resistance mechanism (Kdr-west and Ace-1^R^). Geographic information system was then used to show the distribution of phenotypic and genotypic resistances with Arcgis 10.1 software.

### Study areas

#### Adjohoun

**Figure 1:**
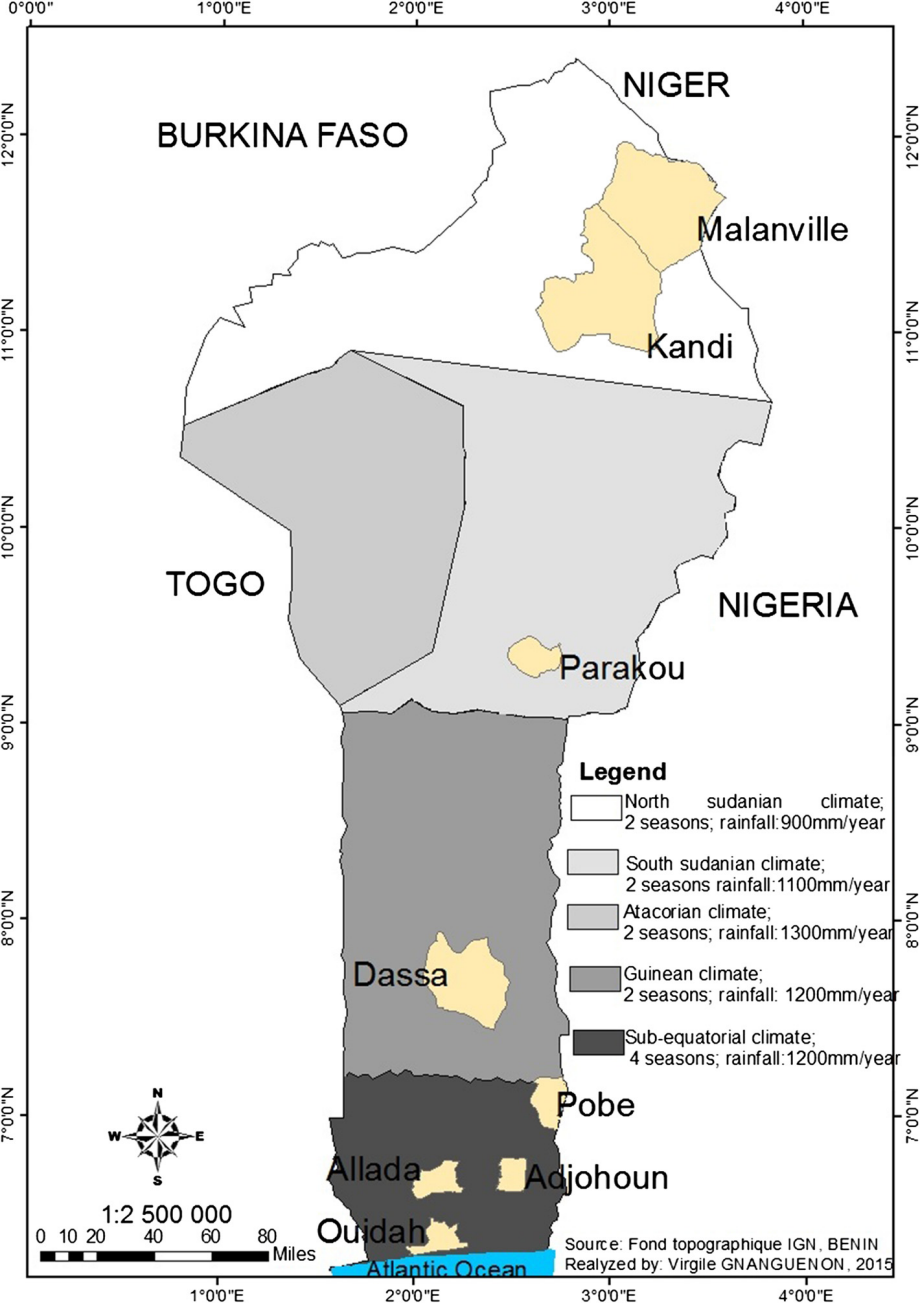
Study sites.

Adjohoun (6°43′12 .76 ″N and 2°29′19.68″E) is located in the center of Oueme County. It is under a subtropical climate with two rainy seasons (a long rainy season from April to July and a short one from September to November) and two dry seasons (a long dry season from December to March and a short from September to August). The annual average rainfall is 1122 mm [18] (Figure 1).

#### Allada

Allada (6°44′37.01″N and 2°8′13.32″E) is located in the north of Atlantique County. The climate is sub-equatorial with two rainy seasons and two dry seasons similar to those of Adjohoun. The annual average rainfall is about 800–1100 mm [16] (Figure 1).

#### Pobe

Pobe (7°49′58.83″N and 2°13′37.60″E) located in southeastern Benin in Plateau County and have a boundary with Nigeria. The climate is sub-equatorial with the same two rainy seasons and two dry seasons as above (characteristics of south Benin). The rainfall varies from 1100-1200 mm per year [17] (Figure 1).

#### Ouidah

Ouidah (6°19′14.48″N and 2°4′0.25″E) belongs to a geographic area called “wet zone”. The climate is sub-equatorial, characterized by two rainy seasons and two dry seasons. The rainfall varies from 950-1150 mm per year [15] (Figure 1).

#### Dassa

Dassa-Zoume (7°49′58.83″N and 2°13′37.60″E) is one of the six districts in Collines County. The climate is Guinean with two rainy seasons and two dry seasons. The highest rainfall is recorded between July and September. The average rainfall is around 1100 mm [21] (Figure 1).

#### Parakou

Regional capital of northern Benin, Parakou (7°49′58.83″N and 2°13′37.60″E) is located in Borgou County. The climate is South Sudanian-characterized by a rainy season (May to October) and a dry season (November-April). The average annual rainfall is 1200 mm. The maximum occurs from July to September [22] (Figure 1).

#### Kandi

Kandi (11°4′58.91″N and 2°13′37.60″E) is located in the center of Alibori County. The climate is North Sudanian characterized by two distinct seasons: a rainy season from April to October and a dry season from November to March. The annual average rainfall varies considerably between 700 and 1400 mm [19] (Figure 1).

#### Malanville

Malanville (11°48′49.06″N and 3°22′58.08″E) is located in the extreme north of Benin in Alibori County. The climate of Malanville is North Sudanian. The average rainfall turns around 750 mm [20] (Figure 1).

### Mosquito collections

*Anopheles gambiae sensu lato* larvae and pupae were collected from natural breeding sites of each district. Mosquito larvae and pupae collected were kept in separated labeled bottles related to each district, transported to the insectaria and maintained at a relative humidity of 72 ± 5% and a temperature of 28 ± 2°C. *An. gambiae s.l* larvae were identified and separated for rearing. Adults were provided with 10% sugar solution. Unfed *An. gambiae s.l* adults, female aged from 2 to 5 days from each district were pooled together to have a sizable mosquito sample (n =150).

### Insecticide susceptibility tests

Mosquitoes were assessed using World Health Organization discriminating dosages with four insecticides: 0.1% Deltamethrin, 0.1% Bendiocarb, 0.25% Pirimiphos méthyl, and 1% Fenitrothion. 20–25 unfed females were exposed to the diagnostic doses of insecticide treated papers for 60 min at 27 ± 1°C and 80% relative humidity. Exposed mosquitoes were introduced into each tube and inspected at different time intervals (10, 15, 20, 30, 45, 60 minutes), ‘Immediate mortality’ (0.1% Bendiocarb, 0.25% Pirimiphos méthyl, and 1% Fenitrothion) and “knocked-down” (for 0.1% Deltamethrin) were recorded. After exposure, mosquitoes were kept in observation tubes and provided with a 10% honey solution. Mosquitoes exposed to untreated papers were used as control. Mortalities were recorded after 24 hours and susceptibility status of mosquito populations was graded according to the World Health Organization protocol [24]. Dead and surviving mosquitoes from assessments were used for molecular analysis.

### Species identification and PCR detection of Knock-down resistance (Kdr) and insensitive acetylcholinesterase-1 (Ace-1^R^) mutations

Around 48 mosquitoes by site were randomly selected from live and dead mosquitoes and subjected to Polymerase Chain Reaction (PCR) for species identification [25]. DNA extracted from specimens of *Anopheles gambiae sensu stricto* were subjected to PCR for identification of ‘*Anopheles coluzzii’* and ‘*Anopheles gambiae*’ [26].

The Polymerase Chain Reaction-Restriction Fragment Length Polymorphism diagnostic test was used to detect the presence of L1014F mutation (Kdr) according to the method described by Martinez-Torres *et al.* [27] and G119S mutation (Ace-1^R^) using the method describe by Weill *et al.* [28].

### Biochemical analysis

Biochemical analysis was performed on 50 mosquitoes stored at 280 μC within 24 h from emergence from each district. These mosquito samples were not exposed to any insecticides prior to biochemical assays. Levels of activity of mixed function oxidases (MFO), non-specific esterases (α and β-esterases), and glutathione S-transferases (GST) were compared in susceptible *An. gambiae* Kisumu and the field populations from each district.

Oxydase activity was assessed using heme-peroxidase assay to identify the elevation in the amount of heme according to the method described by Brogdon *et al.* [29]. Non-specific esterase activity was measured using a-naphtol acetate and b-naphtol acetate and final concentrations were determined at 550 nm [30]. Glutathione- S-transferase (GST) activity was measured in mosquitoes using 200 ml of GSH/CDNB working solution added to each replicate of mosquito homogenate. The kinetic reaction was read at 340 nm immediately for 5 minutes [30].

### Data analysis

World Health Organization criteria [24] were used to determine resistance status of mosquito population as follows:

- **Mortality rate is > 98%:** susceptible mosquito population;
- **Mortality rates ranged between 90 – 98%:** suspected resistance in the mosquito population;
- **Mortality rates < 90%:** resistant mosquito population to the insecticide.

Knock-down and Immediate mortality were very low at 10 minutes and were then analyzed from 15 minutes. Mortality rates of *An. gambiae* populations were compared using Fisher’s exact test. Allelic frequencies of L1014F mutation and G119S mutation were analyzed to assess variability of mutation frequencies across populations. Only Deltamethrin, Knock-down and Ace-1^R^ resistance data were analyzed for Ouidah due to several unsuccessful mosquito larvae collections at this site.

All statistical analysis was performed using R 2.15 software.

## Results

### Effect of Deltamethrin on mosquitoes

**Figure 2:**
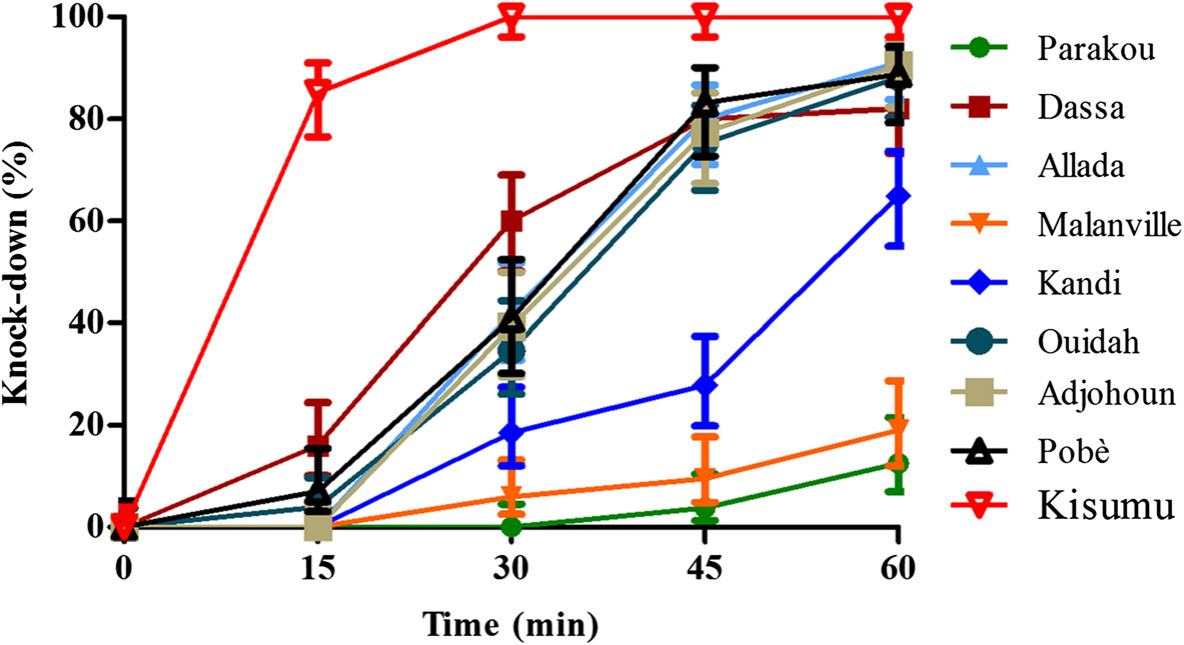
Knock-down rates of mosquitoes due to exposure to Deltamethrin.

The knock-down effect of Deltamethrin on mosquitoes tested was very low (Figure 2). After 15 minutes, the proportion of mosquitoes knocked-down was between 0 and 16% versus 86% for the negative control Kisumu. The effect of Deltamethrin on the different populations after 15 minutes of exposure was similar (p > 0.05). The knock-down effect after 30 minutes of exposure to Deltamethrin varied from 0 to 60% but with a significantly low effect on populations of mosquitoes Parakou, Kandi and Malanville compared to other sites (P < 0.05). At 60 minutes exposure, the knock-down effect of Deltamethrin on mosquito populations tested was less than 80% versus 100% for the susceptible strain Kisumu (control). Parakou and Malanville showed the lowest Knock-down effects (12–19%) after an hour of exposure.

**Figure 3:**
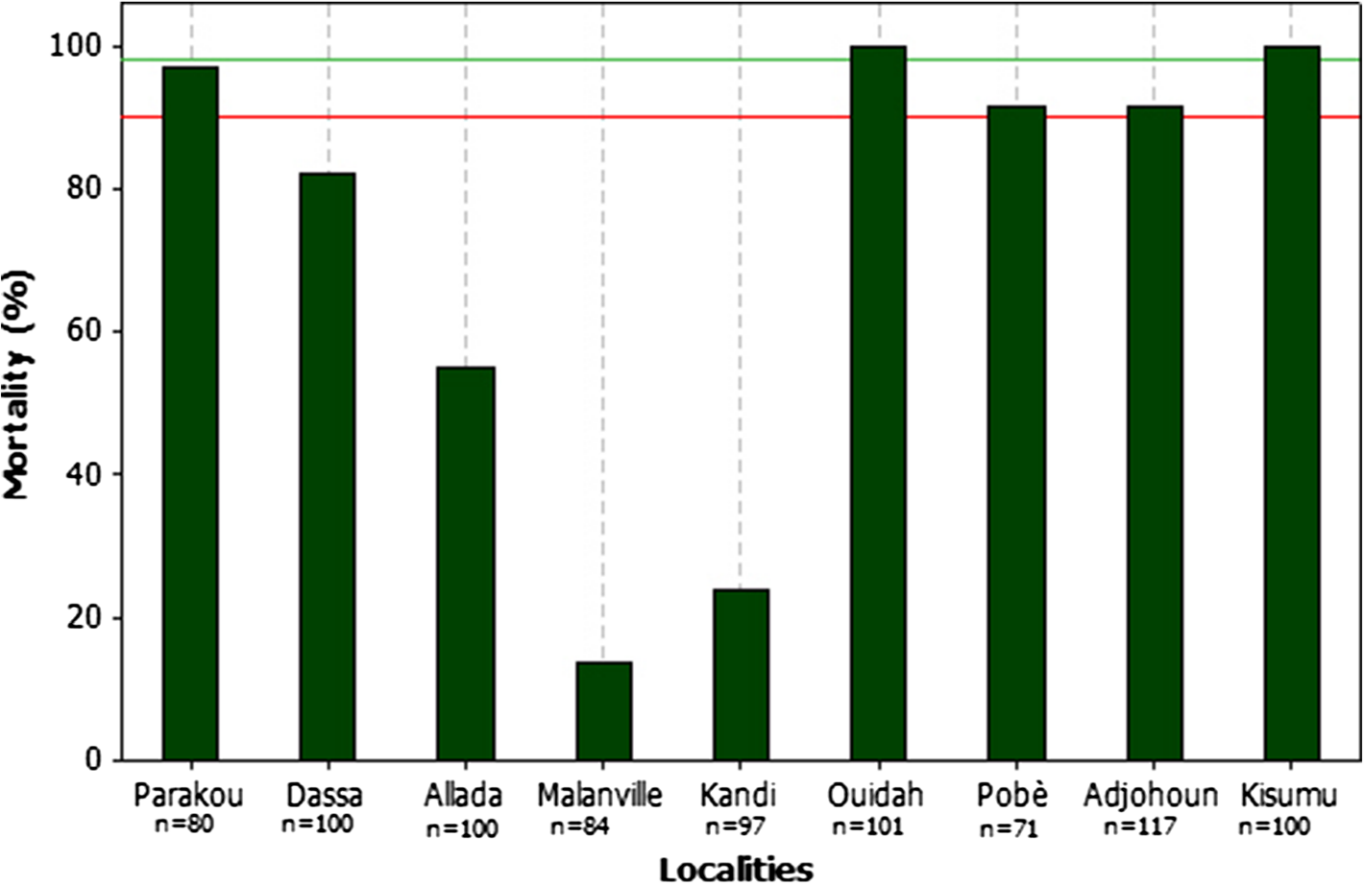
Observed mortalities with Deltamethrin.

Twenty-four hours post-exposure (Figure 3), the highest mortality rate (100%) was observed with mosquito population from Ouidah (versus 100% for Kisumu), suggesting a full susceptibility of this population to Deltamethrin (Figure 3). A suspected resistance to Deltamethrin of mosquito populations from Pobe and Parakou was observed with respective mortalities of 91 and 97%. In Malanville, Kandi, Dassa, Adjohoun and Allada mortalities ranged between 14 and 87%, showing that vectors from these sites were resistant to Deltamethrin.

### Effect of Bendiocarb on mosquitoes

**Figure 4:**
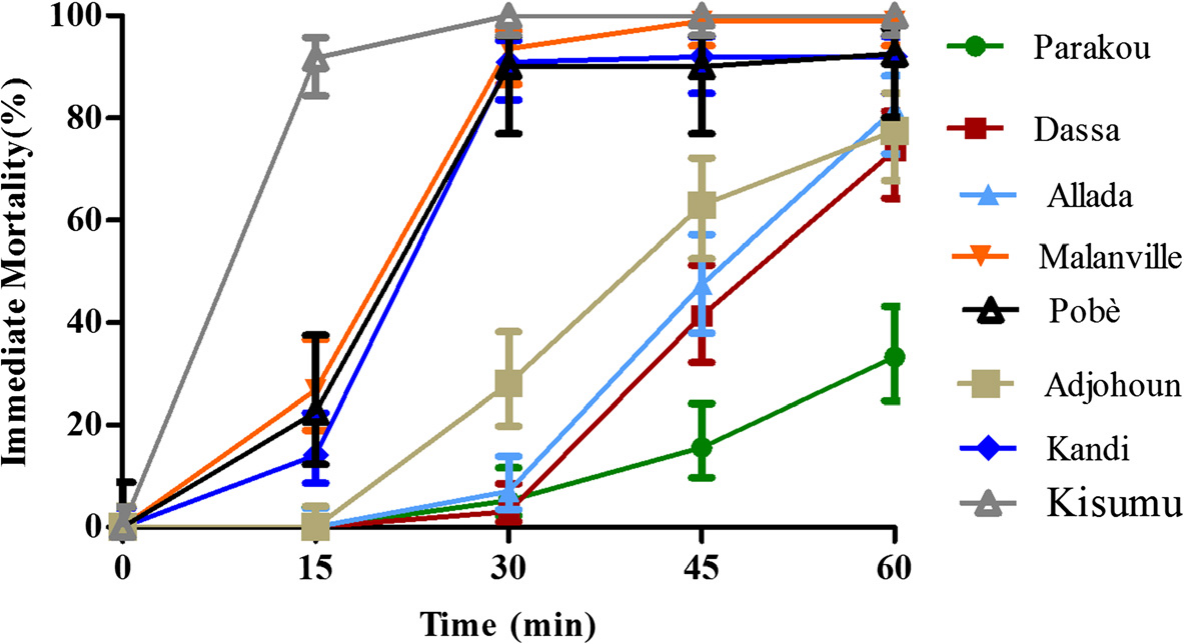
Immediate mortalities observed during mosquitoes exposure to Bendiocarb.

After 15 min, the immediate mortalities induced by Bendiocarb on different population of mosquitoes varied between 0 and 27%. A significant difference was observed between the immediate mortalities induced by Bendiocarb on mosquitoes from Pobè, Kandi and Malanville and those of Parakou, Allada, Dassa and Adjohoun (p < 0.05). The proportion of dead mosquitoes after 30 minutes of exposure to Bendiocarb is from 3 to 93%. After 60 minutes of exposure, approximately 80% of tested mosquitoes are dead except Parakou, Dassa and Adjohoun where the observed mortality is significantly lower (33–77%) (Figure 4).

**Figure 5:**
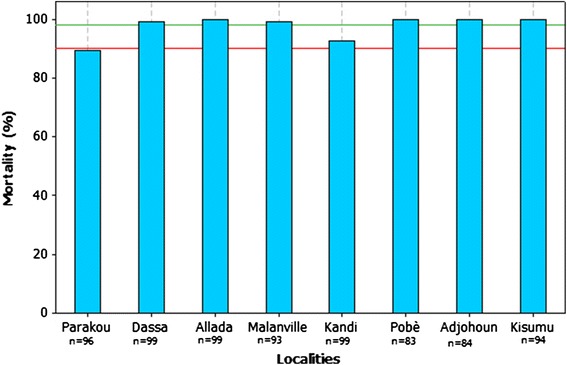
Observed mortalities with Bendiocarb.

Mortality rates observed after 24 hours in Pobè, Dassa, Allada, Malanville and Adjohoun exceeded 98% (Figure 5) versus 100% for Kisumu. At Kandi and Parakou the observed mortality rates were respectively 92.93% [86.12 to 96.53] and 89.53% [81.88 to 94.24]. This shows a reduced susceptibility in mosquito of Kandi and resistance in mosquito of Parakou (Figure 5). These results suggest the emergence of resistance to Bendiocarb from the north of Benin (Figure 5).

### Effect of Pyrimiphos methyl on mosquitoes

**Figure 6:**
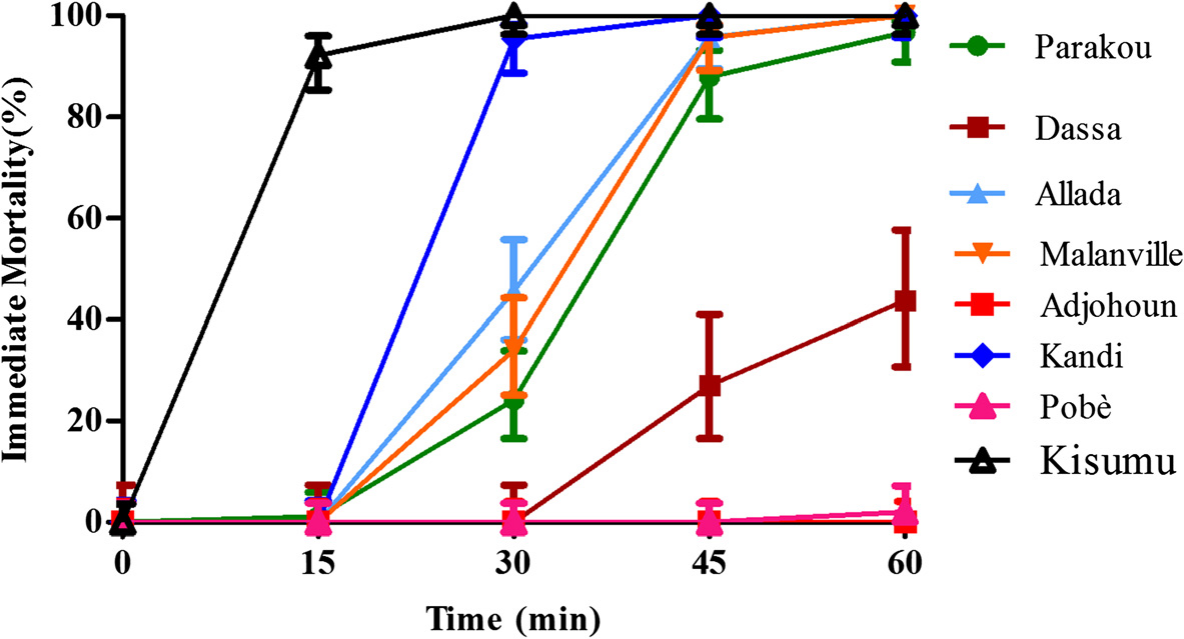
Immediate mortalities observed during mosquitoes exposure to Pyrimiphos methyl.

The immediate mortalities induced by Pirimiphosmethyl varied significantly between different mosquito populations tested (p <0.05) (Figure 6). It is after thirteen minutes that the effect of Pirimiphos methyl was more perceptible on mosquitoes (Figure 6). The observed immediate mortalities range from 24–95% in Allada, Parakou, Kandi and Malanville. But, in Pobè, Adjohoun and Allada, the majority of mosquitoes were still alive (Figure 6). After an hour of exposure, immediate mortality increased in all sites (43–100%) except Pobè and Adjohoun where mortality is almost zero (Figure 6).

**Figure 7:**
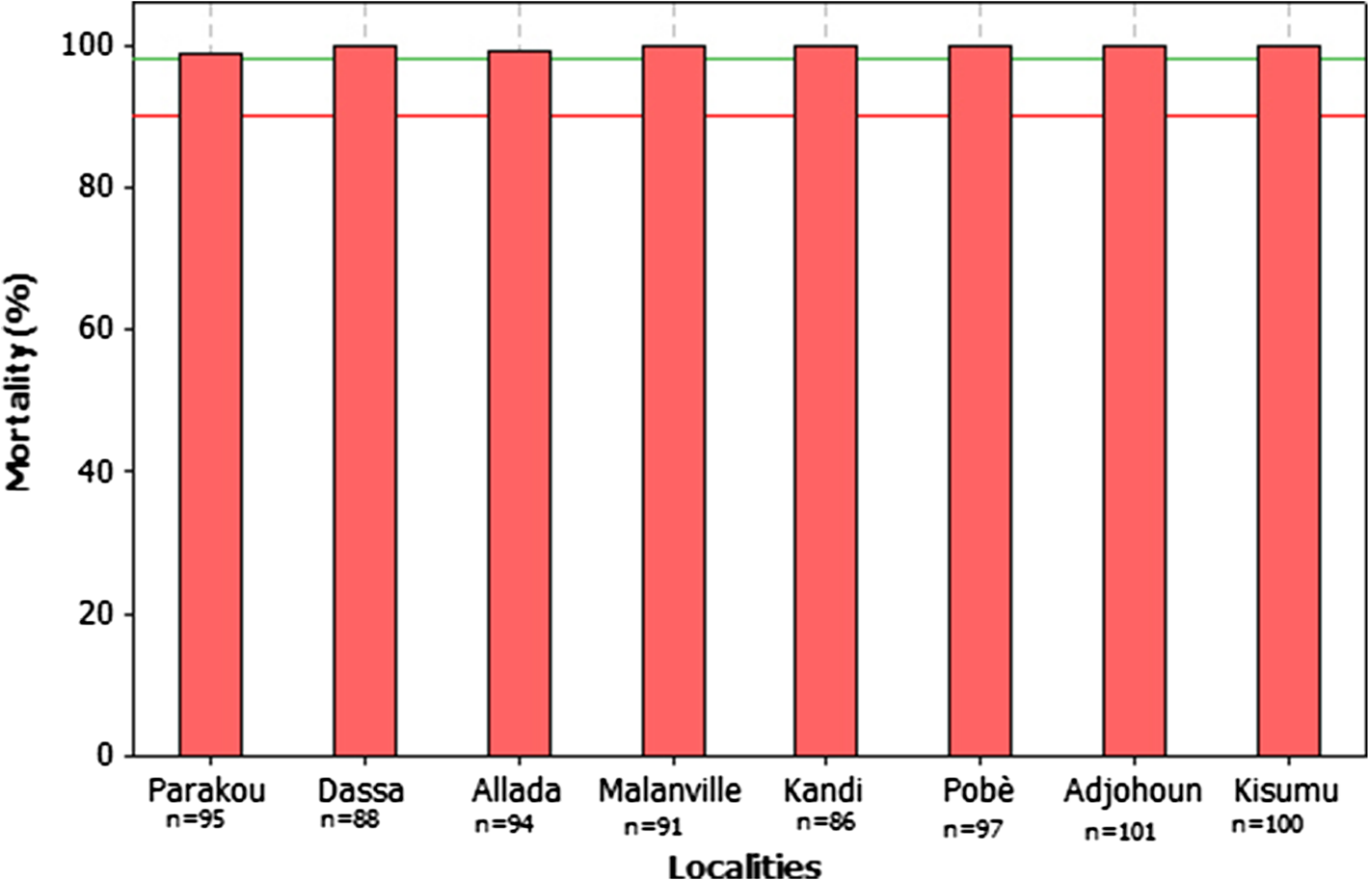
Mortalities observed with Pyrimiphos methyl.

Mortalities noted after 24 hours of observation were 100% suggesting a complete susceptibility of all mosquito populations tested to Pirimiphos methyl (Figure 7).

### Fenitrothion effect on mosquitoes

The effect of Fenitrothion on mosquitoes tested was low and similar between mosquito populations during exposure (Table 1). After 15 and 30 minutes of exposure, the observed immediate mortalities were almost null (0 to 2.40%). After 45 minutes, the immediate effect of Fenitrothion was more evident on mosquito population from Malanville (25.61% [17.40 to 36.00]) and Allada (23% [32, 15 to 15.84]) versus 100% fro Kisumu. At 60 minutes of exposure, immediate mortalities increased to 75% in Malanville, Kandi, Parakou, and Dassa Allada. But, in Pobè and Adjohoun immediate mortalities remained zero.

**Table 1:** **Observed Knock-down rate and mortality with Fenitrothion**

**Localities**	**Number tested**	**Knock-down (%)**					**Mortality (%)**
		**0 min**	**15 min**	**30 min**	**45 min**	**60 min**	
**Parakou**	86	0[0,00-4,28]	0[0,00-4,28]	0[0,00-4,28]	0[0,00-4,28]	24[16,56-34,46]	100
**Dassa**	99	0[0,00-3,74]	0[0,00-3,74]	1,01[0,18-5,5]	3[1,04-8,53]	23[16,01-32,46]	100
**Allada**	90	0[0,00-3,70]	1[0,18-5,45]	1[0,18-5,45]	23[15,84-32,15]	61[69,98-51,2]	100
**Malanville**	82	0[0,00-4,48]	2[0,67-8,46]	2[0,67-8,46]	26[17,4-36]	76[65,31-83,62]	100
**Kandi**	100	0[0,00-3,70]	0[0,00-3,70]	0[0,00-3,70]	0[0,00-3,70]	44[34,67-53,77]	100
**Adjohoun**	95	0[0,00-3,89]	0[0,00-3,89]	0[0,00-3,89]	0[0,00-3,89]	0[0,00-03,89]	100
**Pobè**	93	0[0,00-3,97]	0[0,00-3,97]	0[0,00-3,97]	0[0,00-3,97]	0[0,00-03,97]	100
**Kisumu**	98	0[0,00-3,77]	82[72,83-88,05]	100[96,23-100]	100[96,23-100]	100[96,23-100]	100

min = minutes.

After 24 hours of observation, mortalities varied from 98 to 100% suggesting that all vector populations tested were susceptible to Fenitrothion (Table 1).

**Figure 8:**
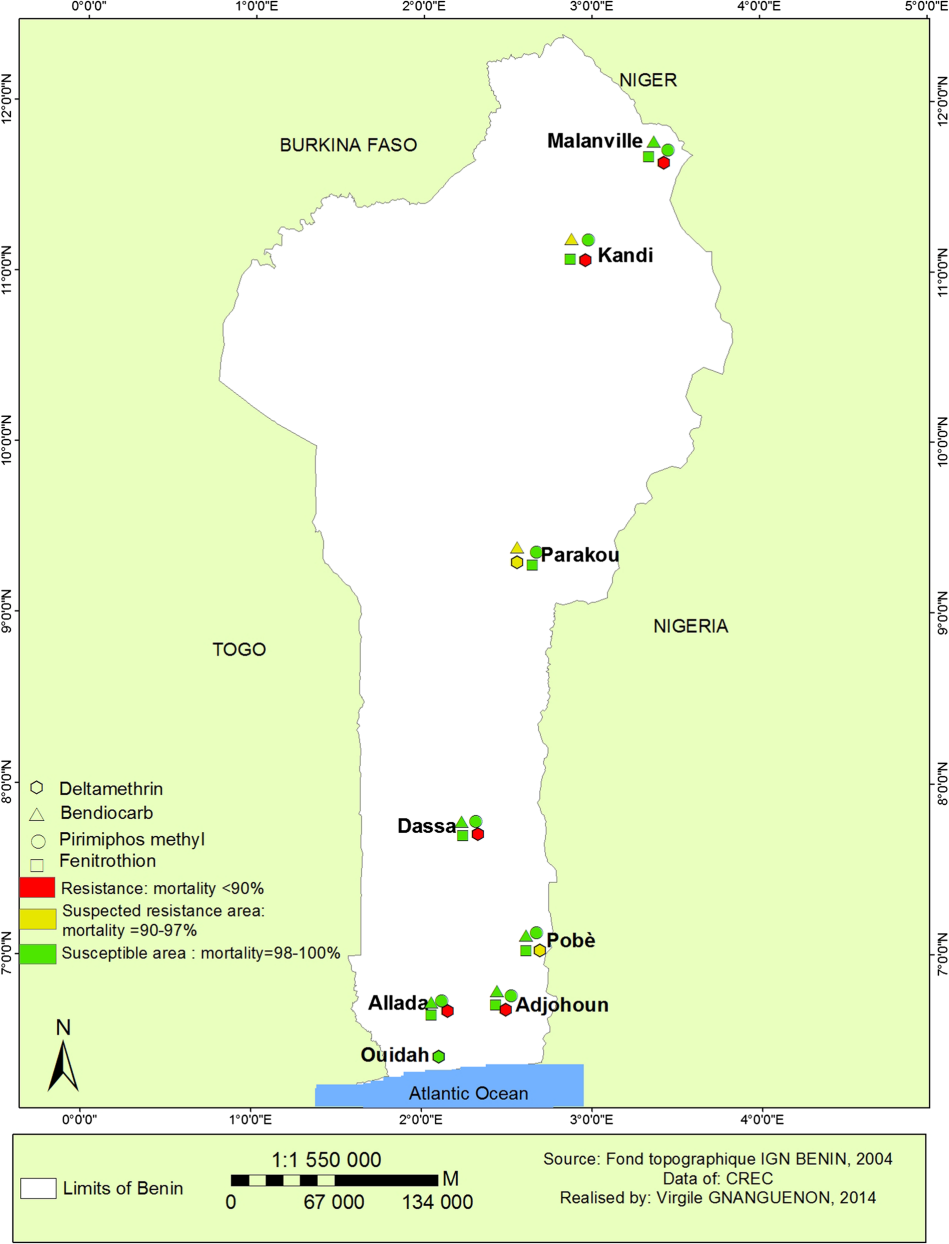
Distribution map of insecticides resistance in Benin.

In summary, Deltamethrin resistance was widely distributed throughout the country (Figure 8). A suspected resistance to Bendiocarb was observed from the northern part of the country while a full susceptibility was observed in the south (Figure 8). A full susceptibility of vector populations to Pirimiphos methyl and Fenitrothion was also from south to north (Figure 8).

### Multiple insecticide resistance mechanisms in *Anopheles gambiae*

Data presented in Table 2 shows the distribution of Knock-down resistance among *An. gambiae* complex species (*An. gambiae, An. arabiensis, An. coluzzi,* and *An. melas*). *An. gambiae, An. arabiensis,* and *An. coluzzi* were identified at Allada, Dassa, Parakou, Malanville, and Kandi but at Ouidah, *An. melas* was found instead of *An. arabiensis* (Table 2). Knock-down resistance frequencies were high (78–93%) in all species and at all sites. No significant difference was observed when comparing Kdr freaquency between *An. gambiae*, *An. Arabiensis*, *An. coluzzi*, and *An. melas* (p > 0.05). RR and RS genotypes of Kdr were found in both dead and survivors but no SS was found.

**Table 2:** **Distribution of Knock-down resistance (Kdr) frequencies between malaria vectors and sites**

**District**	**Species**	**Number**	**RR**	**RS**	**SS**	**F (Kdr)**	**OR (95% CI)**	**p-value**
Allada	*An. gambiae*	14	11	3	0	0,89a	1.00	-
	*An. arabiensis*	7	5	2	0	0,86a	1.39 [0.20-9.44]	1.000
	*An. coluzzi*	20	13	7	0	0,83a	1.77 [0.41-7.53]	0.5072
Dassa	*An. gambiae*	21	17	4	0	0,90a	1.00	-
	*An. arabiensis*	8	5	3	0	0,81a	2.19 [0.43-11.12]	0.381
	*An. coluzzi*	18	15	3	0	0,92a	0.86 [0.18-4.14]	1.00
Parakou	*An. gambiae*	15	13	2	0	0,93a	1.00	-
	*An. arabiensis*	19	15	4	0	0,89a	1.65 [0.28-9.67]	0.6870
	*An. coluzzi*	14	10	4	0	0,86a	2.33 [0.39-13.87]	0.4154
Kandi	*An. gambiae*	22	16	6	0	0,86a	1.00	-
	*An. arabiensis*	14	12	2	0	0,93a	0.49 [0.09-2.60]	0.4705
	*An. coluzzi*	12	11	1	0	0,96a	0.27 [0.03-2.43]	0.4073
Malanville	*An. gambiae*	18	14	4	0	0,89a	1.00	-
	*An. arabiensis*	3	2	1	0	0,83a	1.6 [0.15-17.38]	0.5568
	*An. coluzzi*	27	19	7	1	0,83a	1.6 [0.45-5.65]	0.55141
Ouidah	*An. gambiae*	9	5	4	0	0,78a	1.00	-
	*An. melas*	27	19	8	0	0,85a	0.61 [0.16-2.33]	0.47922
	*An. coluzzi*	11	8	3	0	0,86a	0.55 [0.11-2.87]	0.67983

SS = homozygous susceptible; RS = hybrid resistant and susceptible; RR = homozygous resistant, F = Frequency.

Insensitive acetylcholinesterase-1 mutation (Ace-1^R^) was also identified at Kandi and Parakou at very low frequency (1%). At the other localities this mutation was absent with a null frequency (Table 3). It was not detected in *An. coluzzii*, *An. arabiensis* and *An. melas* species but only in heterozygous form in four surviving *An. gambiae* species (three from Parakou and one from Kandi) that were previously identified with Knock-down resistance mutation (Table 3).

**Table 3:** **Distribution of Ace-1R frequency between species**

**District**	**Species**	**Number**	**RR**	**RS**	**SS**	**Ace-1R**	**p-value**
Allada	*An. gambiae*	14	0	0	14	0	P > 0.999
	*An. arabiensis*	7	0	0	7	0	
	*An. coluzzi*	20	0	0	20	0	
Dassa	*An. gambiae*	21	0	0	21	0	P > 0.999
	*An. arabiensis*	8	0	0	8	0	
	*An. coluzzi*	18	0	0	18	0	
Parakou	*An. gambiae*	15	0	3	12	0.1	0,22
	*An. arabiensis*	19	0	0	19	0	
	*An. coluzzi*	14	0	0	14	0	
Kandi	*An. gambiae*	22	0	1	21	0.002	P > 0.999
	*An. arabiensis*	14	0	0	14	0	
	*An. coluzzi*	12	0	0	12	0	
Malanville	*An. gambiae*	18	0	0	18	0	P > 0.999
	*An. arabiensis*	3	0	0	3	0	
	*An. coluzzi*	27	0	0	0	0	
Ouidah	*An. gambiae*	9	0	0	9	0	P > 0.999
	*An. melas*	27	0	0	27	0	
	*An. coluzzi*	11	0	0	11	0	

SS = homozygous susceptible; RS = hybrid resistant and susceptible; RR = homozygous resistant.

**Figure 9:**
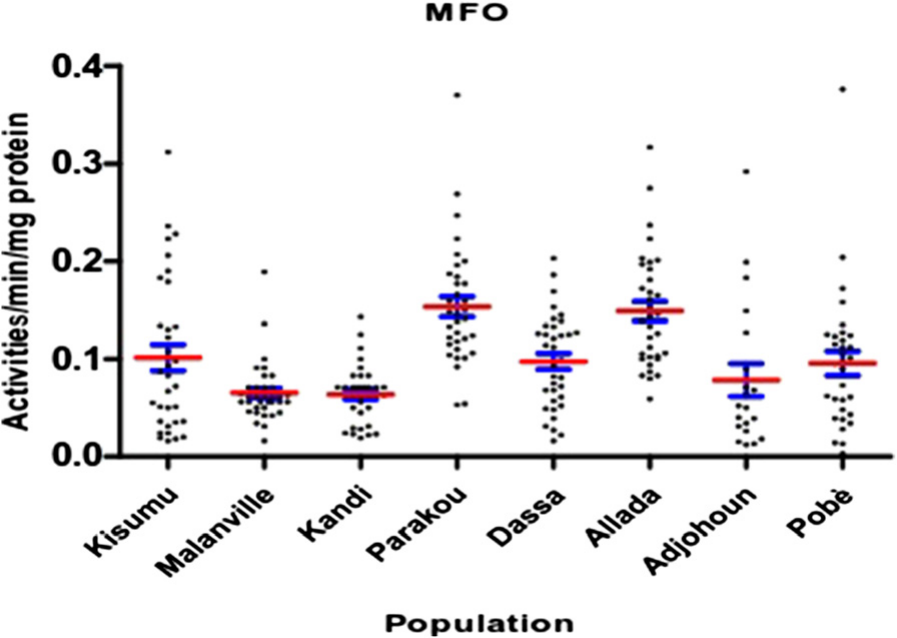
Mono-oxygenase activities in *Anopheles gambiae sensu lato* collected.

Biochemical assays showed significantly high enzymatic activities (MFO, NSE and GST) in some populations of mosquitoes. Figure 9 shows the average level of oxidase activity (MFO) in the different populations tested. The activity of cytochrome P450 was significantly higher in Allada Parakou compared to Kisumu (p < 0.05) (Figure 9).

**Figure 10:**
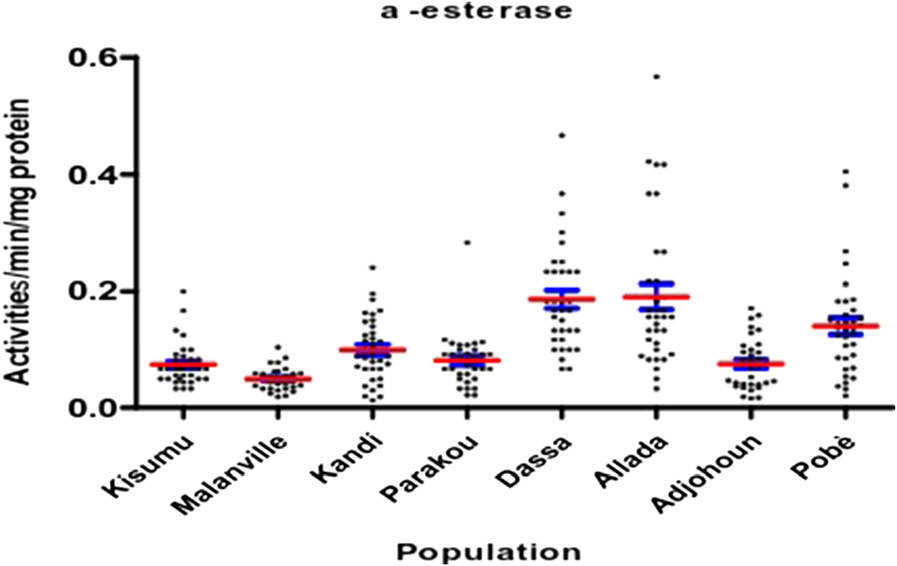
α-esterases activities in *Anopheles gambiae sensu lato* collected.

**Figure 11:**
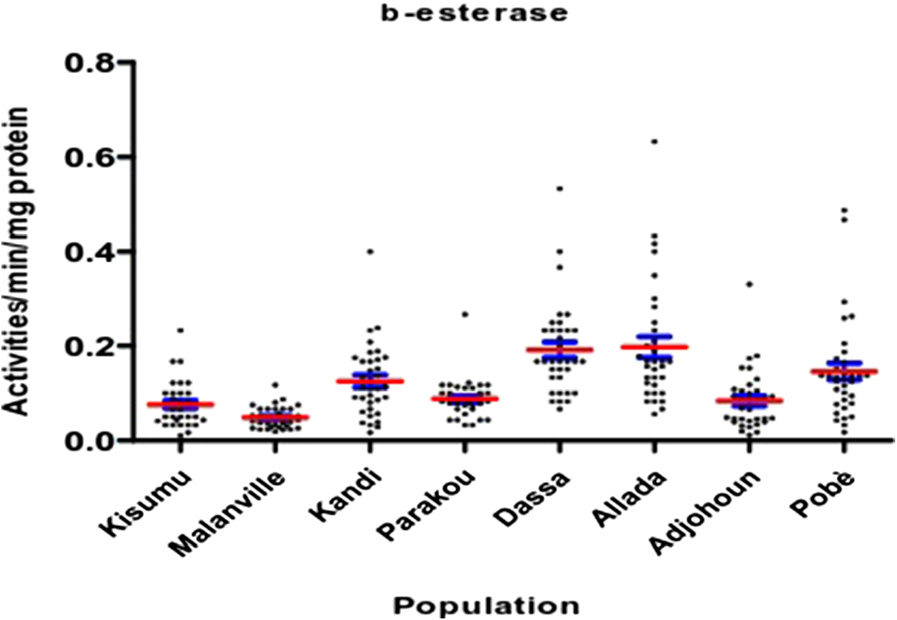
β-esterases activities in *Anopheles gambiae sensu lato* collected.

The activity of non-specific esterase (α and β esterase), was higher in mosquito populations from Kandi, Dassa Allada and Pobè compared to Kisumu (p < 0.05) (Figures 10 and 11).

**Figure 12:**
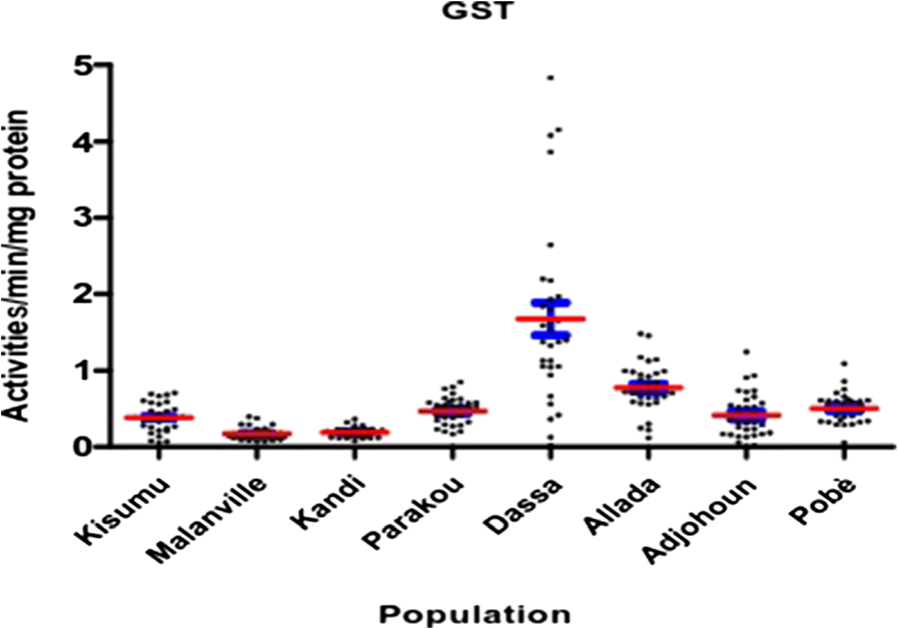
Glutathion-S-transferase activities in *Anopheles gambiae sensu lato* collected.

High activities of the Glutathione-S-transferase were observed in the populations of Dassa Allada and Pobè compared to Kisumu (p < 0.05) (Figure 12).

**Figure 13:**
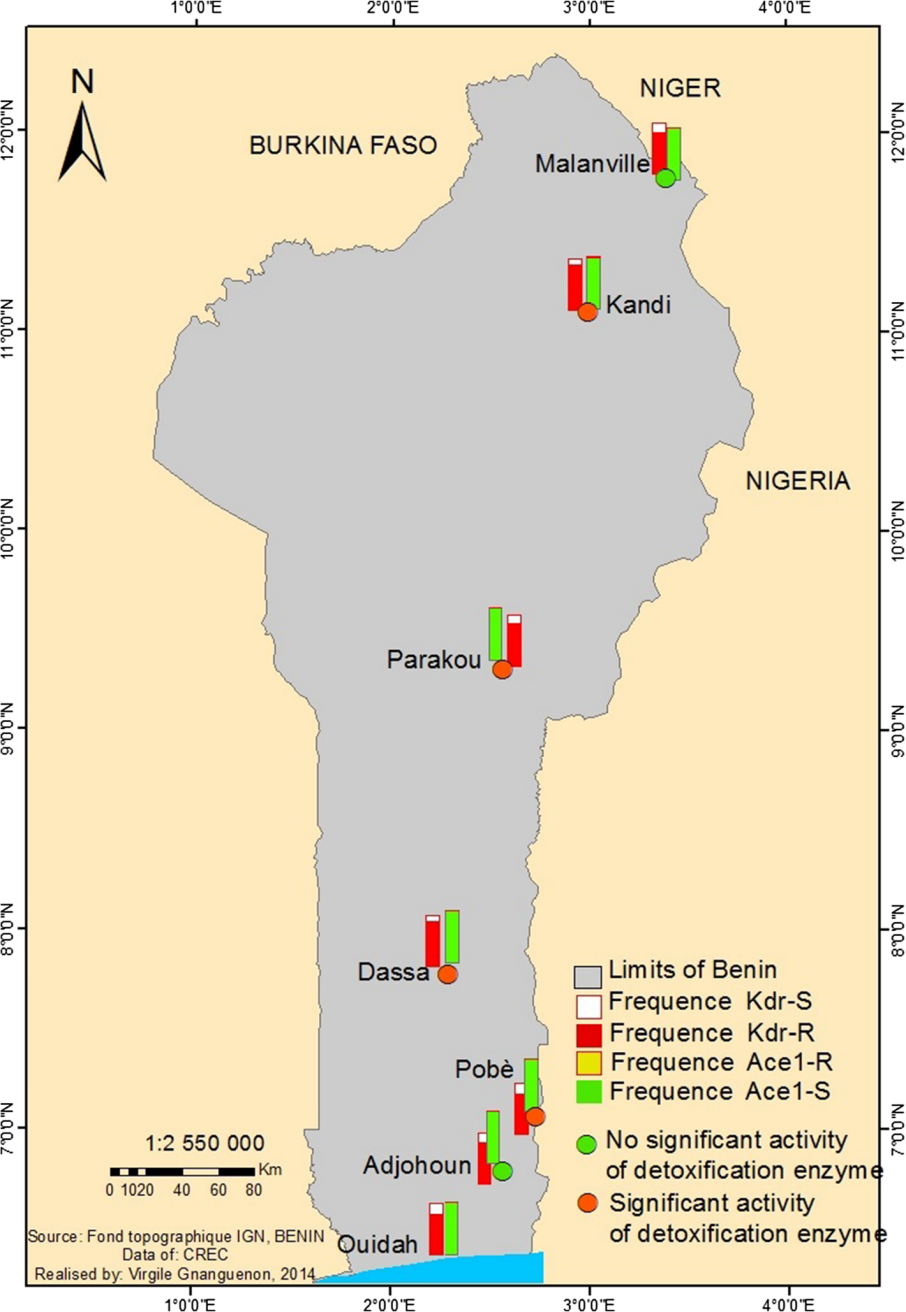
Distribution of resistance mechanism in Benin.

The observed molecular and biochemical resistance mechanisms were mapped to show the distribution of these resistances (Figure 13).

## Discussion

The present study updated data on vectors resistance to the insecticides currently used in vectors control strategies in Benin and shows their distribution. It showed a confirmed resistance to Deltamethrin (pirethrinoid) throughout the country except in Ouidah, an emerging resistance to Bendiorcarb (carbamates) in northern Benin, and a full susceptibility to Pirimiphos methyl and Fenitrothion (organophosphate) with a slow effect of Fenitrothion according to WHO thresholds. These thresholds have no epidemiological meaning because the field performance of the insecticides tested remain high [31-33]. However, they were more likely designed to be the triggers of preventive action by national malaria control program to define efficient strategies to manage insecticide resistance without waiting for indisputable proof of interventions failure [24].

Resistance levels to Deltamethrin varied between localities reflecting variation in resistance selection pressures on different vector populations. Deltamethrin and Permethrin are the most found insecticides on mass-distributed insecticide-treated nets in Benin (from national campaign and routine distributions) with similar coverage rates between regions (74-94%) [34,35]. This should select resistance in the wild mosquito populations [36] and might explain the similar frequencies of Knock-down resistance (Kdr) between regions. In addition, there are also selection pressures generated by the use of the same class of insecticide in agriculture that represent the main activities of certain districts [37-39] and could explain the observed variations. The observed resistance level to Deltamethrin observed in southern Benin was similar to the results previously reported by Djègbè *et al.* [13] and Sovi *et al.* [40].

The Knock-down resistance gene was the main resistance mechanism found in all assessed mosquito populations. It was found at very high frequencies (0.80 in average) in *An. gambiae*, *An. coluzzii*, *An. arabiensis* and *An. melas* populations tested, and was widespread in the country. Their spread could be due to both vector bio-ecology and resistance selection pressure due to the intensive use of public health insecticides in agriculture [41].

In addition to pyrethroid resistance, we observed the emergence of carbamates resistance. This resistance to carbamates was previously reported in Benin by Djogbenou *et al.* [42] and Aïkpon *et al.* [9]. It was associated with very low frequency of insensitive Ace-1 gene detected. This mutation was previously reported by Corbel *et al.* [11], Djogbénou *et al.* [43], Djenontin *et al.* [12], Yadouléton *et al.* [44] and by Aikpon *et al.* [9]. This early spread of Ace-1^R^ mutation should be monitored closely. As the use of Bendiocarb in Indoor Residual Spray is part of a National Malaria Control Program strategy it could increase the frequency of the Ace-1^R^ gene and lead to a widespread of this resistance mutation. It is urgent for this purpose to follow-up monitoring for a better management of this resistance mechanism.

However, even if Kdr and Ace-1R genes confer a significant effect on vector resistance, they do not fully explain the observed vectors resistance level to insecticides because homozygous susceptible subjects (SS) survived to pyrethroids and carbamates exposure suggesting an involvement of other alternative mechanisms such as metabolic resistance mechanisms [45].

To assess the metabolic resistance mechanisms involved in the assessed vector populations, a biochemical approach was used. High activity of glutathione-s-transferase, cytochrome P450 and non-specific esterases were observed in some areas of southern and central Benin. These results were also previously reported in Atacora province [23] and in Cameroon [46]. These enzymes can confer resistance to organophosphates, organochlorines and pyrethroids [47]. They were found with Kdr mutation in the same vector populations and could confer a cross resistance to pyrethroids. The over expression of these enzymes could also explain the slow effect of some organophosphates like Fenitrothion on mosquito populations in absence of insensitive acetylcholinesterase-1.

Ideally, when the resistance is detected for an insecticide, other insecticide categories must be used as part of a dynamic resistance management. However, very few insecticides are currently available for treatment of mosquito nets and indoor residual spray [48,49]. The insecticide change can also lead to higher costs of intervention programs reducing resistance managing options. For example, the change from Bendiocarb (8 US$/house) to Fenitrothion (15.40 US$/house) in indoor residual spray intervention will be 2 times more expensive [50]. In general, the use of insecticides does not create resistance itself, but selects a small proportion of subjects with a genetic mutation that allow them to resist and survive the effects of the insecticide. If this advantage is maintained by a constant use of the same insecticide, the resistant insects will reproduce and the genetic changes that confer resistance are transferred from parents to offspring so that eventually resistant subjects become numerous within the population [51,52]. The spread of resistant subjects will take more time if the resistance gene is rare or present at low frequency. It is also complex and depends on several genetic, biological and operational factors [51]. Biological factors affect the life cycle of the insect (the reproduction rate, the number of generation or offspring, the rate of migration and isolation, etc.), while genetic factors include intrinsic characteristics of the resistance genes (mono function of polygenic resistance, domination, genetic cost and the interaction of genes) [53]. Operational factors concern the insecticide treatment itself, including the method and frequency of application, dosage and the residual activity of the insecticide and the level of insecticide coverage.

In theory, the resistance management of insecticide resistance should be performed by alternating vector control methods based on the use of insecticides and non-insecticidal methods. In practice, most non-insecticidal methods work well in experimental trials but become difficult when programs intensify their long-term use (operational) [54]. Then, operationally, the simplest way of resistance management should involve insecticides management. The implementation of management strategies that may delay selection and spread of resistance to insecticides is therefore the main gateway for vector control programs. Another alternative is to find other insecticide formulations that will enhance the choice possibilities of insecticides.

This data will enable the National Malaria control programme to determine what insecticide to use and where. They should implement a rapid pro-active response to avoid vectors resistance to Pirimiphosmethyl and delay the spread of Bendiocarb resistance. Alternative non-insecticidal methods should be used, wherever possible, to delay insecticide resistance.

## Conclusion

This study shows the resistance profile of malaria vectors to different categories of insecticides used for vector control in Benin. A widespread malaria vector resistance to pyrethroid and an early emergence of carbamates resistance in the northern region of the country were observed. However, vectors were fully susceptible to organophosphates including Pirimiphos methyl and Fenitrothion but with a rapid susceptibility to the effect of Pirimiphos methyl.

This useful information could help policy-makers to better plan insecticide resistance management. The most convenient option is based on the appropriate use of insecticides. The moment of insecticide use, method of use (combined or single), the frequency and duration of use need to be planned accordingly to delay the spread of vectors resistance.
